# Supplementary material for: Connecting multiple microenvironment proteomes uncovers the biology in head and neck cancer
Source: Nat Commun. 2022 Nov 7;13:6725. doi: 10.1038/s41467-022-34407-1 (PMC9640649; doi:10.1038/s41467-022-34407-1)
Supplement: Supplementary file 1 — Supplementary Information [file 41467_2022_34407_MOESM1_ESM.pdf]

Supplementary Information

Connecting multiple microenvironment proteomes uncovers the biology in head and neck cancer

Ariane F. Busso-Lopes[1], Leandro X. Neves[1], Guilherme A. Câmara[1], Daniela C. Granato[1], Marco Antônio M. Pretti[2], Henry Heberle[3], Fábio M.S. Patroni[4], Jamile Sá[1], Sami Yokoo[1], César Rivera[1,5,6], Romênia R. Domingues[1], Ana Gabriela C. Normando[1,5], Tatiane De Rossi[1], Barbara P. Mello[7], Nayane A. L. Galdino[8], Bianca A. Pauletti[1], Pammela A. Lacerda[9], André Afonso N. Rodrigues[10], André Luis M. Casarim[10], Reydson A. de Lima- Souza[5], Ingrid I. Damas[11], Fernanda V. Mariano[11], Kenneth J. Gollob[8,12], Tiago S. Medina[8], Nilva K. Cervigne[9], Ana Carolina Prado-Ribeiro[5,13], Thaís Bianca Brandão[13], Luisa L. Villa[7,13], Miyuki Uno[13], Mariana Boroni[2], Luiz Paulo Kowalski[14,15], Wilfredo Alejandro González-Arriagada[16,17], Adriana F. Paes Leme[1,*]

[1]Laboratório Nacional de Biociências - LNBio, Centro Nacional de Pesquisa em Energia e Materiais - CNPEM, Campinas, SP, 13083-100, Brazil; [2]Laboratório de Bioinformática e Biologia Computacional, Divisão de Pesquisa Experimental e Translacional, Instituto Nacional do Câncer - INCA, Rio de Janeiro, RJ, 20231-050, Brazil; [3]Instituto de Ciências Matemáticas e de Computação, Universidade de São Paulo - USP, São Carlos, SP, 13566-590, Brazil; [4]Centro de Biologia Molecular e Engenharia Genética – CBMEG, Universidade Estadual de Campinas - UNICAMP, Campinas, SP, 13083-887, Brazil; [5]Departamento de Diagnóstico Oral, Faculdade de Odontologia de Piracicaba, Universidade Estadual de Campinas - UNICAMP, Piracicaba, SP, 13414-903, Brazil; [6]Departamento de Ciencias Básicas Biomédicas, Facultad de Ciencias de la Salud, Universidad de Talca - UTALCA, Talca, Maule, Chile; [7]Departamento de Radiologia e Oncologia, Faculdade de Medicina, Universidade de São Paulo – USP, São Paulo, SP, 01246-903, Brazil; [8]Centro Internacional de Pesquisa - CIPE, A.C. Camargo Cancer Center, São Paulo, SP, 01508-010, Brazil; [9]Departamento de Clínica Médica, Faculdade de Medicina de Jundiaí - FMJ, Jundiaí, SP, 13202-550, Brazil; [10]Departamento de Cirurgia de Cabeça e Pescoço, Faculdade de Medicina de Jundiaí - FMJ, Jundiaí, SP, 13202-550, Brazil; [11]Departamento de Patologia, Faculdade de Ciências Médicas, Universidade Estadual de Campinas – UNICAMP; Campinas, SP, 13083-887, Brazil; [12]Hospital Israelita Albert Einstein, São Paulo, SP, 05652- 900, Brazil; [13]Instituto do Câncer do Estado de São Paulo - ICESP, Faculdade de Medicina, Universidade de São Paulo - USP, São Paulo, SP, 01246-000, Brazil; [14]Departamento de Cirurgia de Cabeça e Pescoço e Otorrinolaringologia, A.C. Camargo Cancer Center, São Paulo, SP, 01509-900, Brazil; [15]Departamento de Cirurgia de Cabeça e Pescoço, Faculdade de Medicina, Universidade de São Paulo - USP, São Paulo, SP, 01246-903, Brazil; [16]Facultad de Odontología, Patología Oral y Maxilofacial, Universidad de Los Andes, Santiago, Chile; [17]Centro de Investigación e Innovación Biomédica, Universidad de Los Andes, Santiago, Chile; [*]Corresponding author. E-mail: adriana.paesleme@lnbio.cnpem.br.

Table of Contents

Supplementary Figures 1-7
Supplementary References

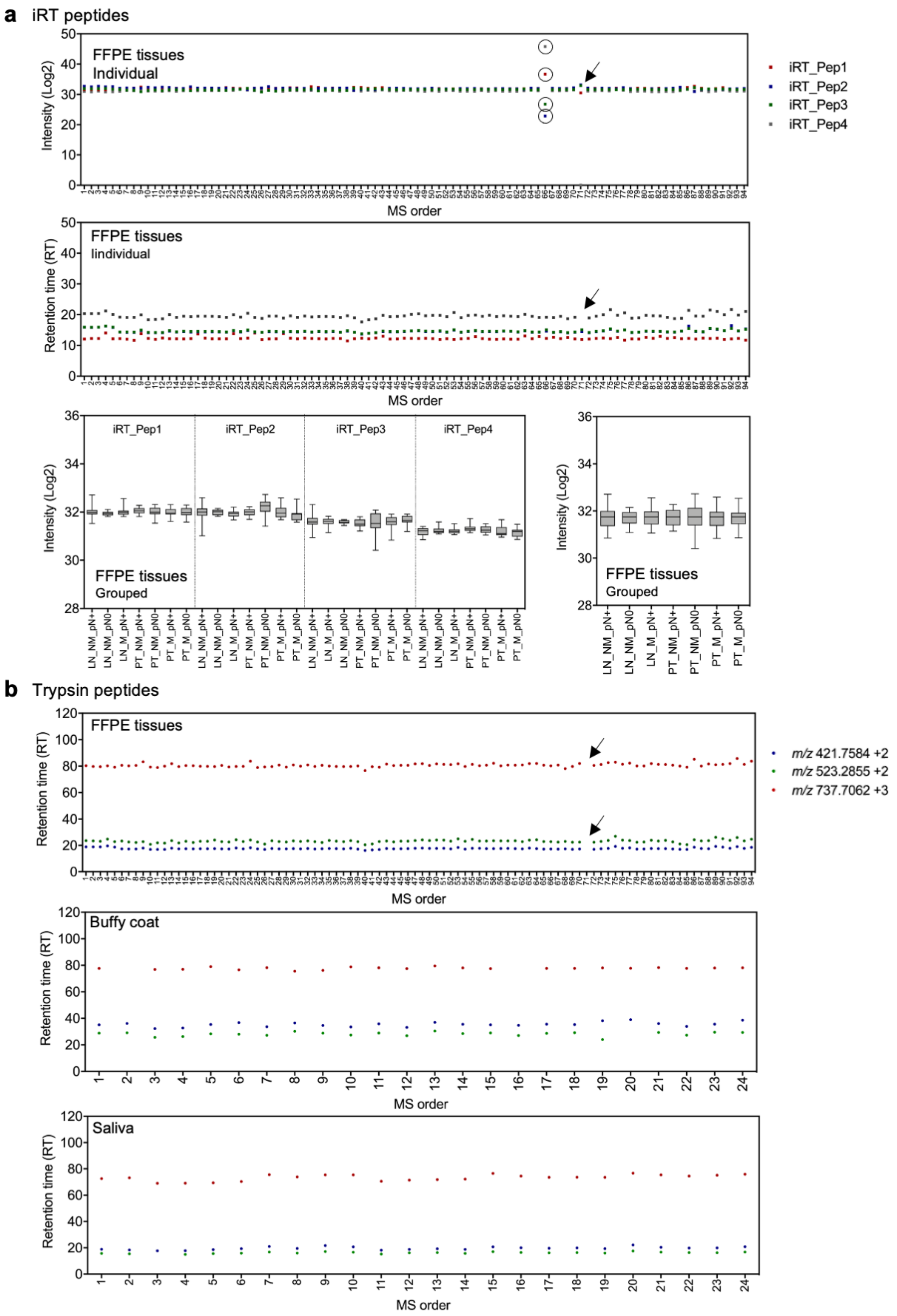
a
iRT peptides
FFPE tissues
Individual
Intensity (Log2)
MS order
iRT_Pep1
iRT_Pep2
iRT_Pep3
iRT_Pep4
FFPE tissues
Iindividual
Retention time (RT)
MS order
iRT_Pep1
iRT_Pep2
iRT_Pep3
iRT_Pep4
FFPE tissues
Grouped
Intensity (Log2)
LN_NM_pN+
LN_NM_pN0
LN_M_pN+
PT_NM_pN+
PT_NM_pN0
PT_M_pN+
PT_M_pN0
FFPE tissues
Grouped
b
Trypsin peptides
FFPE tissues
Retention time (RT)
MS order
m/z 421.7584 +2
m/z 523.2855 +2
m/z 737.7062 +3
Buffy coat
Retention time (RT)
MS order
Saliva
Retention time (RT)
MS order

Supplementary Figure 1 iRT and trypsin quality control for 142 tissue and fluid samples evaluated by mass spectrometry in the discovery phase (LC-MS/MS, DDA). a Intensities and retention times for 4 iRT peptides (50 fmol) used to monitor deviations in mass spectrometry runs for 94 individual FFPE samples (27 malignant cells from primary tumor, 13 malignant cells from lymph nodes, 27 non-malignant cells from primary tumor, and 27 non-malignant cells from lymph node) and for each group of comparisons. Black circles indicate discrepant intensities for the four iRT peptides in case 66 (malignant cells from primary tumor 4417), and the arrows show absent intensity and retention time for iRT_Pep4 in case 71 (malignant cells from primary tumor 2875). The two samples were excluded from further analysis due to the inconsistent profile. No deviations were observed in the chromatographic pattern among groups. **b** Retention times for 3 trypsin autolysis peaks (*m*/*z* 421.7584, +2; *m*/*z* 523.2855, +2; *m*/*z* 737.7062, +3) used to monitor deviations in sample preparation and LC-mass spectrometry runs for the 94 FFPE tissues (description above), 24 buffy coats, and 24 saliva samples, totalizing 142 runs. Black arrows indicate sample 71 that was missing peaks for the three trypsin peptides evaluated. The sample was excluded from proteomics analysis (sample 2875 – malignant cells from primary tumor). Due to the exclusion of two samples with inconsistent profile, data from the 140 out of 142 runs were used in subsequent analysis. FFPE: Formalin-Fixed Paraffin-Embedded tissue sample, PT_M: primary tumor – malignant cells, PT_NM: primary tumor – non-malignant cells, LN_M: lymph node – malignant cells, LN_NM: lymph node – non-malignant cells.

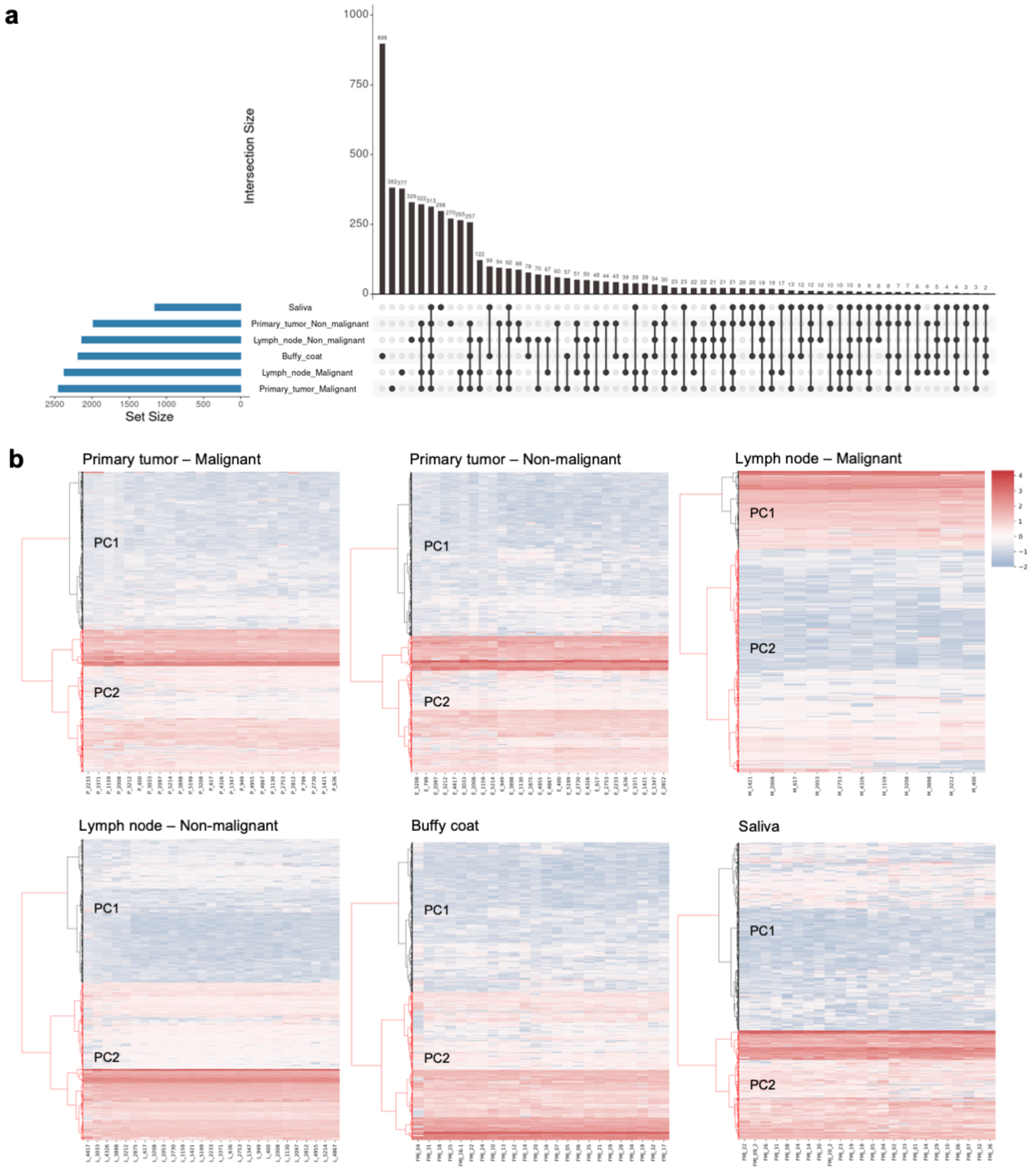

Supplementary Figure 2 Characterization of tissues and fluids according to the proteomic profile in a 59-HNSCC cohort. a Upset plot presenting shared and exclusive proteins for the six HNSCC sites that were evaluated. Proteomic data acquired for each site were run independently in MaxQuant software. **b** Heat maps revealing the proteins clusters (PC1 and PC2) that were identified for cell populations from tissues and fluids. PC groups were generated using the Ward's method based on Bray-Curtis distance for 25 primary tumor – malignant samples (2,444 proteins), 27 primary tumor – non-malignant samples (1,984 proteins), 24 buffy coats (2,188 proteins), 13 lymph node – malignant samples (2,308 proteins), 27 lymph node – non-malignant samples (2,137 proteins), and 24 saliva samples (1,154 proteins). Source data are provided as a Source Data file.

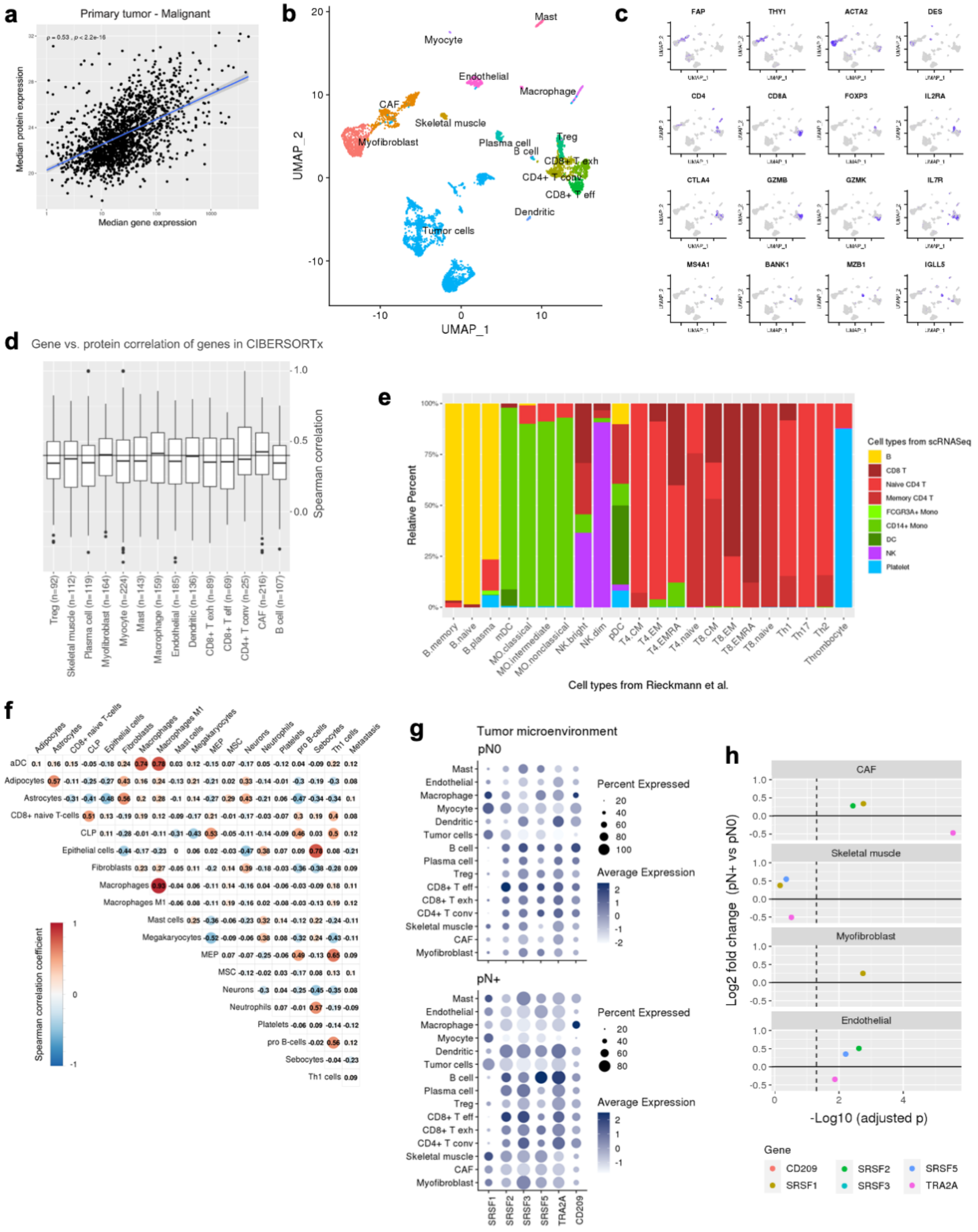

Supplementary Figure 3 Association of the proteome landscape with immunity. a Spearman correlation coefficients generated by comparing the proteomes from HNSCC malignant cells from our dataset to the RNASeq data of tumors from TCGA ($\rho = 0.53$; $p \leq 2.2E\text{-}16$; two-sided Spearman's correlation test). The line represents a linear regression curve, and the shaded region indicates 95% confidence interval for the correlation. **b-c** Annotation of cell populations in the HNSCC scRNASeq dataset using the standard workflow from Seurat. **c** shows expression of cluster-defining genes used for annotations in **b**. **d** Spearman correlation coefficients generated by comparing the protein

abundance and gene expression of 1,840 genes used in the HNSCC signature matrix of CIBERSORTx version 1.0 [1] (two-sided Spearman's correlation test). Data were recovered from an expression atlas of 28 healthy tissues. The median correlation score (0.4) is shown as a horizontal line and the number of correlation coefficients plotted are presented in the graph for each cell type. Boxplots show the median (central line), the 25–75% interquartile range (IQR) (box limits), and the ±1.5×IQR (whiskers). **e** Proteome deconvolution using LFQ intensities from 22 cell types characterized by Rieckmann et al. [2] in CIBERSORTx version 1.0 using a reference matrix built from PBMC scRNASeq. Each column depicts a population described by Rieckmann et al. [2] and the colors represent the respective cell type assigned by CIBERSORTx using the scRNASeq reference matrix. Bars are shown as relative values. Cells were named as in the referenced manuscript. **f** Spearman correlation coefficients generated for the comparison of nodal status (last column) and immune populations. RNASeq data from HNSCC patients from TCGA with information of nodal metastasis were used to identify cell signatures with the xCell version 1.0 algorithm. Correlations with $p \leq 0.05$ are presented (two-sided Spearman's correlation test). **g** Expression of the six microenvironment targets in immune populations from HNSCC that were identified by scRNASeq [1] in pN+ and pN0 primary tumors. **h** Average fold change and differential expression in non-immune populations of the six targets from the tumor microenvironments selected for verification (pN+ vs. pN0; two-sided Wilcoxon test followed by Benjamini-Hochberg correction). The dashed lines represent a *p* threshold of 0.05.

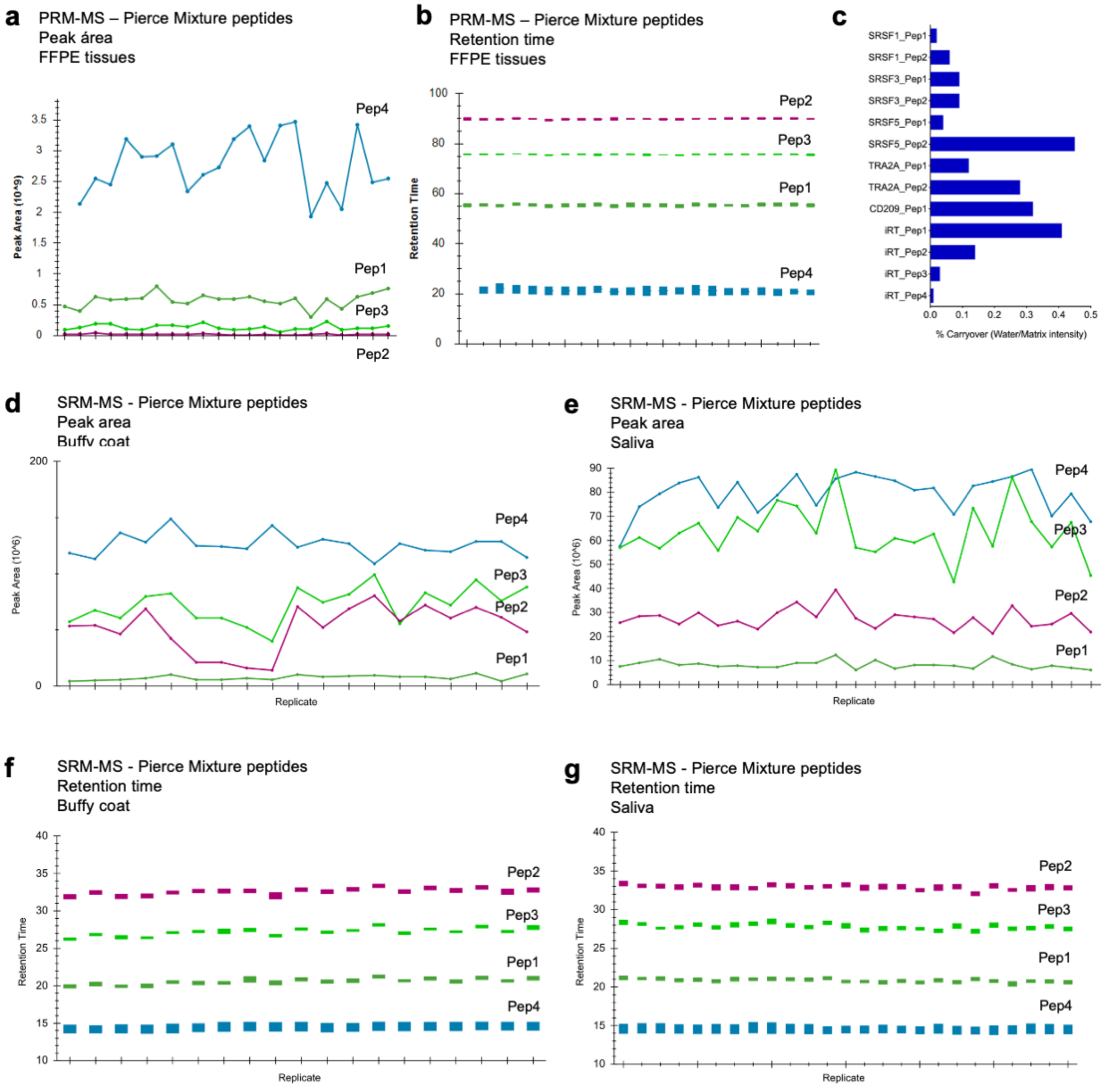

Supplementary Figure 4 Quality control of PRM-MS and SRM-MS experiments. a-b Peak areas **(a)** and retention times **(b)** for iRT mixture (44 fmol) obtained from mass spectrometry runs using PRM-MS. **c** Frequency of carryover for heavy peptides from the microenvironment biomarkers SRSF1, SRSF3, SRSF5, TRA2A, and CD209 (14.4 to 320 fmol) and for 4 iRT peptides (32 fmol) (peptide intensity in water/peptide intensity in buffy coat matrix) using SRM-MS. **d-e** Peak areas for iRT mixture (32 fmol) obtained from mass spectrometry runs in buffy coat (**d**) and saliva (**e**) samples using SRM-MS. **f-g** Retention time for iRT mixture (32 fmol) obtained from mass spectrometry runs in buffy coat (**f**) and saliva (**g**) samples using SRM-MS.

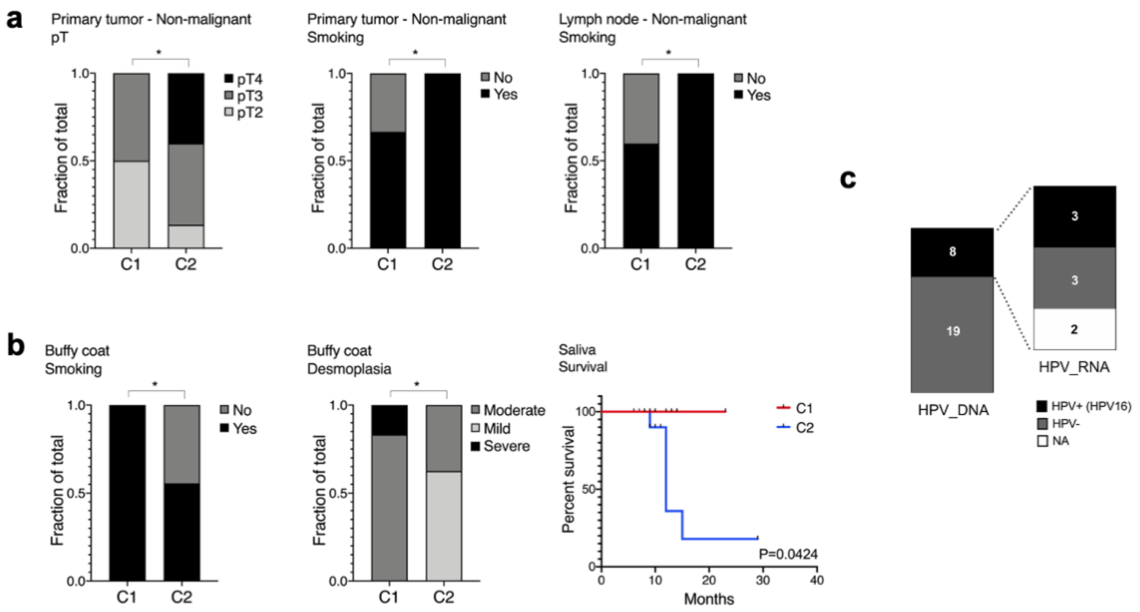

Supplementary Figure 5 Association of the proteome landscape with clinic-pathological data. a Clinico-pathological features from HNSCC patients that were significantly associated with patient clusters C1 and C2 for tissues (n = 27 patients; $p \leq 0.05$; two-sided Fisher's exact test). *$p \leq 0.05$. **b** Clinical-pathological features from HNSCC patients that were significantly associated with patient clusters C1 and C2 for buffy coat (n = 24 patients) and saliva (n = 24 patients) samples ($p \leq 0.05$; two-sided Fisher's exact test and two-sided log-rank test). **c** Evaluation of HPV DNA and RNA in primary tumor tissues (n = 27 patients) from HNSCC patients. NA: not available. *$p \leq 0.05$. Source data are provided as a Source Data file.

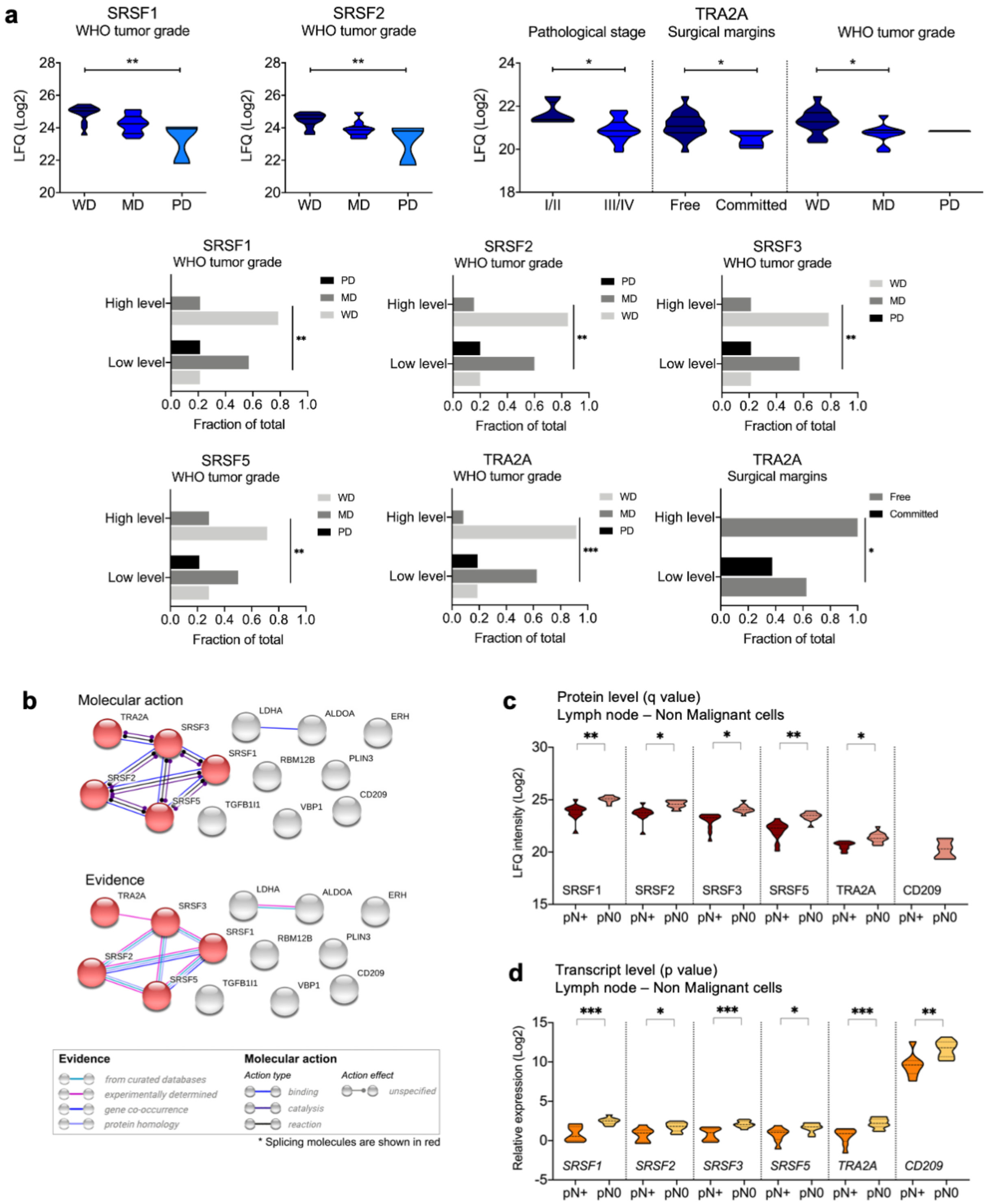

Supplementary Figure 6 Characterization of nodal metastasis biomarkers detected in the lymph node non-malignant cells from 27 HNSCC patients. a Clinico-pathological features significantly associated with the abundance of SRSF1, SRSF2, SRSF3, SRSF5, and TRA2A proteins (n = 27 patients; $p \leq 0.05$; two-sided unpaired Student's t-test, ANOVA, and two-sided Fisher's exact test). High and low levels of proteins were defined using the mean intensity as the cut-off. *$p \leq 0.05$, **$p \leq 0.01$, ***$p \leq 0.001$. **b** Protein interaction networks for the proteins associated with lymph node metastasis. **c** Log2 LFQ intensity for SRSF1, SRSF2, SRSF3, SRSF5, TRA2A, and CD209 in lymph node tissues from HNSCC patients as determined by DDA (n = 27 patients, 13 pN+ and 14 pN0;

pN+ vs pN0; $q \leq 0.05$; two-sided Student's t-test followed by Benjamini-Hochberg correction). CD209 was detected only in pN0 samples. *$q \leq 0.05$, **$q \leq 0.01$. **d** Relative expression of *SRSF1, SRSF2, SRSF3, SRSF5, TRA2A,* and *CD209* in lymph node tissues from HNSCC patients as determined by RT-qPCR (n = 19 patients, 9 pN+ and 10 pN0; pN+ vs pN0; $p \leq 0.05$; two-sided unpaired Student's t-test). *$p \leq 0.05$, **$p \leq 0.01$, ***$p \leq 0.001$. WD: well differentiated tumor, MD: moderately differentiated tumor, PD: poorly differentiated tumor, AUC: area under the curve. Source data are provided as a Source Data file.

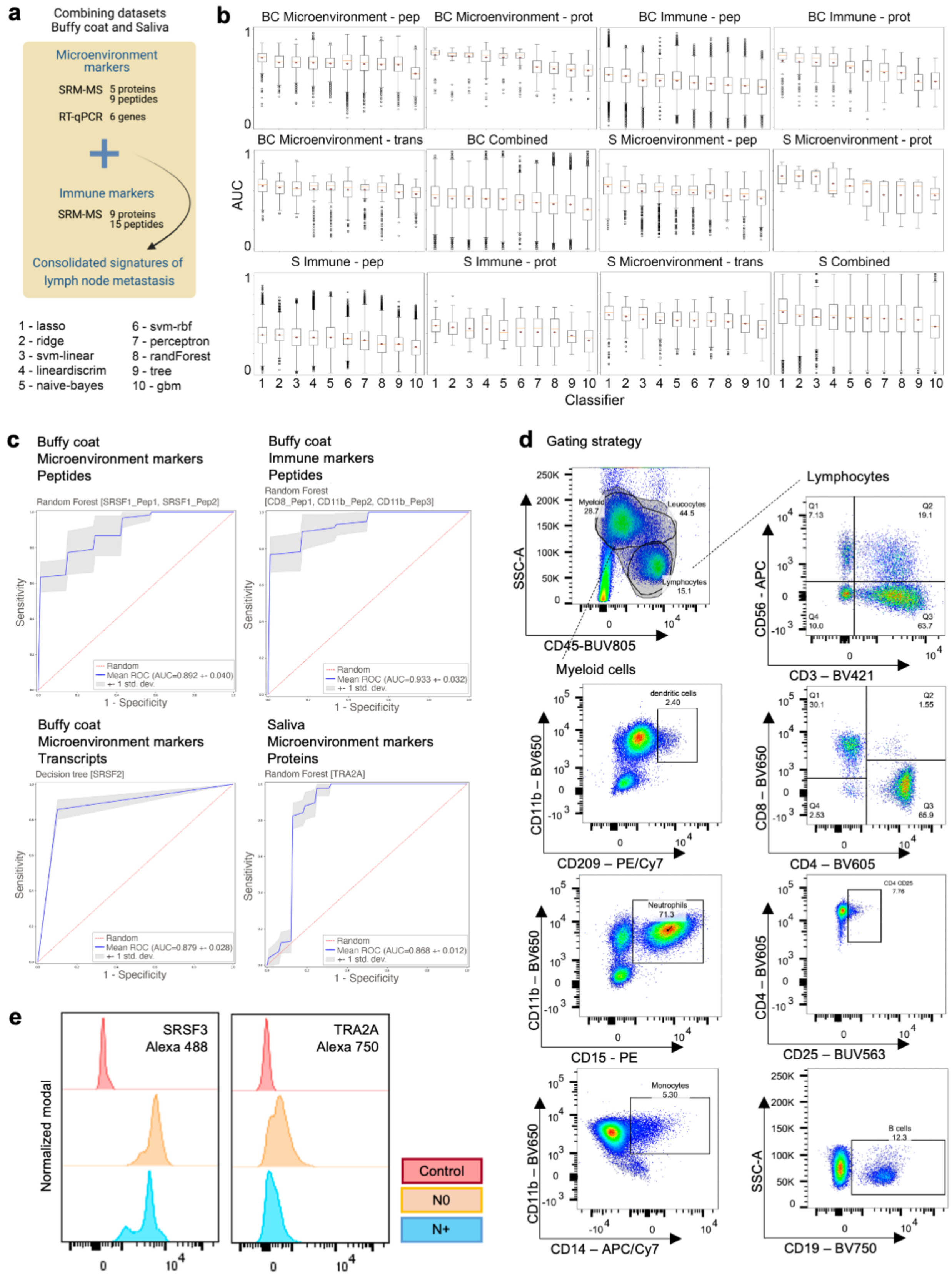

Supplementary Figure 7 Characterization of signatures associated with metastasis according to the machine learning analysis. a Experimental approach to define the best consolidated lymph node metastasis signatures using ML. Created with BioRender.com. **b** AUC distribution per classifier of all signatures identified using ML analysis of buffy coat and saliva datasets. Circles indicate data points that were located outside of the whiskers of the box plots (outliers). Details about the AUCs

plotted for each classifier are available through the Panorama repository at the link https://panoramaweb.org/16fpvB.url. Boxplots show the median (central line), the 25–75% interquartile range (IQR) (box limits), and the ±1.5×IQR (whiskers). **c** ROC curves indicating the top-1 pairs <Si, Cj> for peptides, proteins, or transcripts in individual buffy coat and saliva datasets after filtering for ROC AUC ≥ 0.85 and $p \leq 0.05$ for permutation test (n = 19 patients, 7 pN+ and 12 pN0, for the analysis of microenvironment markers – peptides and immune markers – peptides in buffy coat samples; n = 24 patients, 10 pN+ and 14 pN0, for the analysis of microenvironment markers – transcripts in buffy coat samples; n = 25 patients, 16 pN+ and 9 pN0, for the analysis of microenvironment markers – proteins in saliva samples). Shaded region indicates 95% confidence interval for the AUC. **d** Flow cytometry gating strategy for immune populations from buffy coat samples. Total leukocytes were first gated on a side scatter (SSC-A)/CD45 plot to define lymphocytes and myeloid cells (upper left). The lymphocytes were then gated on the CD19+ (B cells), CD56+CD3- (NK cells) and CD56-CD3+ (T lymphocytes) populations (left). These were further gated on the CD4+, CD8+ and CD4+CD25+ (activated T lymphocytes) subsets (left). The myeloid cells were separated by CD11b expression and further phenotyped according to CD209 (dendritic cells), CD15 (neutrophils) and CD14 (monocytes) expression (right). **e** Histogram of the flow cytometry analysis showing the expression of SRSF3 and TRA2A in buffy coat samples from all N+ and N0 HNSCC patients (10 N+; 10 N0). Two PBMC samples from HNSCC patients were included as controls for surface markers and the expression of SRSF3 and TRA2A is shown in red. AUC: area under the curve; BC: buffy coat; S: saliva; Pep: peptides; Prot: proteins; Trans: transcripts.

Supplementary References

1 Puram, S. V. *et al.* Single-Cell Transcriptomic Analysis of Primary and Metastatic Tumor Ecosystems in Head and Neck Cancer. *Cell* **171**, 1611-1624.e1624, doi:10.1016/j.cell.2017.10.044 (2017).

2 Rieckmann, J. C. *et al.* Social network architecture of human immune cells unveiled by quantitative proteomics. *Nat Immunol* **18**, 583-593, doi:10.1038/ni.3693 (2017).
