# Supplementary material for: Connecting multiple microenvironment proteomes uncovers the biology in head and neck cancer
Source: Nat Commun. 2022 Nov 7;13:6725. doi: 10.1038/s41467-022-34407-1 (PMC9640649; doi:10.1038/s41467-022-34407-1)
Supplement: Supplementary file 3 — Description of Additional Supplementary Files [file 41467_2022_34407_MOESM3_ESM.pdf]

Description of Additional Supplementary Files

File Name: Supplementary Data 1
Description: Samples and main clinical features of HNSCC patients included in this study.

File Name: Supplementary Data 2
Description: Differentially abundant proteins between pN+ and pN0 samples across multiple sites.

File Name: Supplementary Data 3
Description: Functional annotation of proteins that were differentially abundant between lymph nodes and primary sites for malignant and non-malignant samples.

File Name: Supplementary Data 4
Description: SRM-MS, PRM-MS, and RT-qPCR data for all peptides or transcripts quantified in tissues and fluids.

File Name: Supplementary Data 5
Description: Metastasis signatures using machine learning in protein and transcript datasets from multiple sites.

File Name: Supplementary Data 6
Description: Splicing factors associated with lymph node metastasis in HNSCC samples.

File Name: Supplementary Data 7
Description: Oligonucleotides and peptides selected for RT-qPCR, PRM-MS, and SRM-MS experiments.

File Name: Supplementary Data 8
Description: Flow cytometry analysis of SRSF3 and TRA2A proteins in buffy coat samples from HNSCC patients.

File Name: Supplementary Data 9
Description: External datasets used in this study.
