# Supplementary material for: Connecting multiple microenvironment proteomes uncovers the biology in head and neck cancer
Source: Nat Commun. 2022 Nov 7;13:6725. doi: 10.1038/s41467-022-34407-1 (PMC9640649; doi:10.1038/s41467-022-34407-1)
Supplement: Supplementary file 13 — Reporting Summary [file 41467_2022_34407_MOESM13_ESM.pdf]

nature portfolio

Corresponding author(s): Adriana F. Paes Leme

Last updated by author(s): October 20, 2022

Reporting Summary

Nature Portfolio wishes to improve the reproducibility of the work that we publish. This form provides structure for consistency and transparency in reporting. For further information on Nature Portfolio policies, see our Editorial Policies and the Editorial Policy Checklist.

Statistics

For all statistical analyses, confirm that the following items are present in the figure legend, table legend, main text, or Methods section.

n/a	Confirmed	
☐	☒	The exact sample size (*n*) for each experimental group/condition, given as a discrete number and unit of measurement
☐	☒	A statement on whether measurements were taken from distinct samples or whether the same sample was measured repeatedly
☐	☒	The statistical test(s) used AND whether they are one- or two-sided<br>*Only common tests should be described solely by name; describe more complex techniques in the Methods section.*
☐	☒	A description of all covariates tested
☐	☒	A description of any assumptions or corrections, such as tests of normality and adjustment for multiple comparisons
☐	☒	A full description of the statistical parameters including central tendency (e.g. means) or other basic estimates (e.g. regression coefficient) AND variation (e.g. standard deviation) or associated estimates of uncertainty (e.g. confidence intervals)
☐	☒	For null hypothesis testing, the test statistic (e.g. *F*, *t*, *r*) with confidence intervals, effect sizes, degrees of freedom and *P* value noted<br>*Give P values as exact values whenever suitable.*
☒	☐	For Bayesian analysis, information on the choice of priors and Markov chain Monte Carlo settings
☐	☒	For hierarchical and complex designs, identification of the appropriate level for tests and full reporting of outcomes
☐	☒	Estimates of effect sizes (e.g. Cohen's *d*, Pearson's *r*), indicating how they were calculated

Our web collection on statistics for biologists contains articles on many of the points above.

Software and code

Policy information about availability of computer code

Data collection	The mass spectrometry DDA proteomics data were collected using Xcalibur version 2.1 (Thermo Fisher), SRM-MS data were acquired with MassLynx version 4.2 (Waters), PRM-MS data were collected using Xcalibur version 4.4 (Thermo Fisher), and RT-qPCR data information was acquired with 7500 software version 2.3.
Data analysis	MaxQuant version 1.5.8.0; https://www.maxquant.org<br>Perseus version 1.3.0.4; https://maxquant.net/perseus/<br>Skyline version 19.1; https://skyline.ms/project/home/software/Skyline/<br>Skyline version 21.2; https://skyline.ms/project/home/software/Skyline/<br>MSstats version 3.13.6; https://bioconductor.org/packages/MSstats/<br>R version 3.6.2; https://www.R-project.org/<br>R version 4.0.0; https://www.R-project.org/<br>GraphPad Prism version 8.2.1; www.graphpadprism.com<br>IBM SPSS Statistics version 28.0; https://www.ibm.com/analytics/spssstatistics-software<br>fastcluster version 1.1.27; https://CRAN.R-project.org/package=fastcluster<br>SciPy version 1.6; https://docs.scipy.org/doc/scipy/reference/release.1.0.0.html<br>seaborn version 0.11.1; https://pypi.org/project/seaborn/<br>GSEApy version 0.9.18; https://pypi.org/project/gseapy/<br>Python version 3.6; https://www.python.org/<br>Python version 3.7; https://www.python.org/<br>FactoMineR version 1.34; https://CRAN.R-project.org/package=FactoMineR<br>Intervene version 0.6.1; https://asntech.shinyapps.io/intervene/<br>ShinyGO version 0.61; http://bioinformatics.sdstate.edu/go/

UpSet version 1.4.0; https://gehlenborglab.shinyapps.io/upsetr/
STRING version 11.0; https://string-db.org
MassLynx version 4.2; https://www.waters.com/
Primer-BLAST accessed in Oct 2018 and Jun 2022; https://www.ncbi.nlm.nih.gov/tools/primer-blast/
IDT Oligoanalyzer version 3.1; https://www.idtdna.com/pages/tools/oligoanalyzer
xCell version 1.0; http://xcell.ucsf.edu/
CIBERSORTx version 1.0; https://cibersortx.stanford.edu/
Seurat version 4.0.0; https://CRAN.R-project.org/package=Seurat
GEOquery version 2.56.0; https://bioconductor.org/packages/GEOquery/
TCGAbiolinks version 2.16.0; https://bioconductor.org/packages/TCGAbiolinks/
sklearn programming kit version 0.23.1; https://scikit-learn.org/
Hmisc version 4.4-0; https://CRAN.R-project.org/package=Hmisc
dplyr version 1.0.0; https://CRAN.R-project.org/package=dplyr
tibble version 3.0.1; https://CRAN.R-project.org/package=tibble
data.table version 1.13.0; https://CRAN.R-project.org/package=data.table
readxl version 1.3.1; https://CRAN.R-project.org/package=readxl
SummarizedExperiment version 1.18.2; https://bioconductor.org/packages/SummarizedExperiment/
ggpubr version 0.3.0; https://CRAN.R-project.org/package=ggpubr
ggplot2 version 3.3; https://CRAN.R-project.org/package=ggplot2
FlowJo version 10.8; https://www.flowjo.com

For manuscripts utilizing custom algorithms or software that are central to the research but not yet described in published literature, software must be made available to editors and reviewers. We strongly encourage code deposition in a community repository (e.g. GitHub). See the Nature Portfolio guidelines for submitting code & software for further information.

Data

Policy information about availability of data

All manuscripts must include a data availability statement. This statement should provide the following information, where applicable:

- Accession codes, unique identifiers, or web links for publicly available datasets
- A description of any restrictions on data availability
- For clinical datasets or third party data, please ensure that the statement adheres to our policy

The mass spectrometry proteomics data generated in this study are available at ProteomeXchange via the PRIDE partner repository and Panorama repository. DDA proteomics data are available at ProteomeXchange with the dataset identifier PXD027780 (http://proteomecentral.proteomexchange.org/cgi/GetDataset?ID=PXD027780). SRM-MS data and machine learning results are available through the Panorama repository at the link https://panoramaweb.org/16fpvB.url and ProteomeXchange dataset identifier PXD027984 (http://proteomecentral.proteomexchange.org/cgi/GetDataset?ID=PXD027984). PRM-MS data are available at https://panoramaweb.org/0UwcEI.url in the Panorama repository and ProteomeXchange dataset identifier PXD036311 (http://proteomecentral.proteomexchange.org/cgi/GetDataset?ID=PXD036311).

The following public RNASeq and proteomic data were downloaded and analyzed in this study: scRNASeq data of tumors and lymph nodes from 18 HNSCC patients (Gene Expression Omnibus, dataset identifier GSE103322, https://www.ncbi.nlm.nih.gov/geo/query/acc.cgi?acc=GSE103322) 5, scRNASeq data of a PBMC sample from a health donor (https://support.10xgenomics.com/single-cell-gene-expression/datasets/1.1.0/pbmc3k), scRNASeq and proteomic data of an atlas of 28 health tissues (RNA-Seq data: Array Express, dataset identifier E-MTAB-2836, https://www.ebi.ac.uk/biostudies/arrayexpress/studies/E-MTAB-2836; Mass-spectrometry based proteomic data: ProteomeXchange Consortium, dataset identifier PXD010154, http://proteomecentral.proteomexchange.org/cgi/GetDataset?ID=PXD010154) 21, RNASeq data of tumors from a 500 HNSCC patient-cohort (TCGA; https://portal.gdc.cancer.gov), RNASeq data of tumors from a 428 HNSCC patient-cohort (TCGA; https://portal.gdc.cancer.gov), and proteomic data from 22 human PBMC subpopulations (ProteomeXchange Consortium, data set identifier PXD004352, http://proteomecentral.proteomexchange.org/cgi/GetDataset?ID=PXD004352) 22. A list of proteins with their respective annotated gene ontology (GO) biological processes was retrieved from Uniprot (https://www.uniprot.org). The remaining data are available within the Article, Supplementary Information or Source Data file. Source Data are provided with this paper.

Field-specific reporting

Please select the one below that is the best fit for your research. If you are not sure, read the appropriate sections before making your selection.

☒ Life sciences ☐ Behavioural & social sciences ☐ Ecological, evolutionary & environmental sciences

For a reference copy of the document with all sections, see nature.com/documents/nr-reporting-summary-flat.pdf

Life sciences study design

All studies must disclose on these points even when the disclosure is negative.

Sample size	No statistical method was used to calculate the sample sizes in the current study. HNSCC tissues and fluids were included based on the availability of clinical samples and lymph node metastasis information at the time of the study. Sample sizes were considered sufficient based on previous experience of our group with clinical samples to obtain statistical significance. We included 184 samples collected from a 93-patient cohort with HNSCC that was randomly selected from October 2009 to December 2021. The discovery cohort consisted of 59 HNSCC patients from whom 27 FFPE primary tumors, 27 FFPE lymph nodes, 24 blood, and 24 saliva samples were collected and used in discovery proteomics (DDA). The verification cohort comprised an 83-patient group, and 23 FFPE tissues, 47 blood, and 33 saliva samples were used in the PRM-MS, SRM-MS, RT-qPCR, and flow cytometry experiments. The samples were obtained from the Valparaiso University (Chile), Faculty

	of Medicine of Jundiai (Brazil), ICESP (Brazil), and A.C. Camargo Hospital (Brazil) acording to the institutional availability. For more details, please see Supplementary Data 1.
Data exclusions	Two primary tumor samples from malignant cells were excluded due to inconsistent detection of quality control peptide precursor ions of iRT and trypsin in samples from patients 2875 and 4417 (Supplementary figure 1). Two primary tumor FFPE tissues with low signal-to-noise ratios were excluded from PRM-MS analysis (1159 and 2008).
Replication	DDA, SRM-MS, PRM-SM and flow cytometry data were acquired in one replicate and RT-qPCR samples were tested in duplicates, with consistent data across the replicates.
Randomization	Samples were allocated into the clinical groups pN+ and pN0 based on pathological information. Mass spectrometry runs (DDA, SRM-MS and PRM-MS) were randomized in the R environment.
Blinding	For samples processing, data acquisition and data analysis, such as clustering and PCA, investigators were blinded to all clinical information.

Reporting for specific materials, systems and methods

We require information from authors about some types of materials, experimental systems and methods used in many studies. Here, indicate whether each material, system or method listed is relevant to your study. If you are not sure if a list item applies to your research, read the appropriate section before selecting a response.

Materials & experimental systems

n/a	Involved in the study	
☐	☒	Antibodies
☒	☐	Eukaryotic cell lines
☒	☐	Palaeontology and archaeology
☒	☐	Animals and other organisms
☐	☒	Human research participants
☒	☐	Clinical data
☒	☐	Dual use research of concern

Methods

n/a	Involved in the study	
☒	☐	ChIP-seq
☐	☒	Flow cytometry
☒	☐	MRI-based neuroimaging

Antibodies

Antibodies used

1. Alexa Fluor® 488 anti-SRSF3; Abcam; Cat# ab223455, clone EPR16976, 1:20 dilution
2. Rabbit pAB anti-TRA2A; Sigma-Aldrich; Cat# HPA054018, polyclonal, 1:100 dilution
3. PE/Cy7 anti-human CD209 (DC-SIGN); Biolegend; Cat# 330113, clone 9E9A8, 1:20 dilution
4. Brilliant Violet 421™ anti-human CD3; Biolegend; Cat# 300433, clone UCHT1, 1:300 dilution
5. APC/Cyanine7 anti-human CD14; Biolegend; Cat# 367107, clone 63D3, 1:200 dilution
6. BUV805 Mouse Anti-Human CD45; BD Horizon; Cat# 612891, clone HI30, 1:500 dilution
7. BV605 Mouse Anti-Human CD4; BD Horizon; Cat# 565998, clone SK3, 1:300 dilution
8. BV650 Mouse Anti-Human CD8; BD Horizon; Cat# 563821, clone RPA-T8, 1:500 dilution
9. BUV563 Mouse Anti-Human CD25; BD Horizon; Cat# 612918, clone 2A3, 1:500 dilution
10. APC Mouse Anti-Human CD56; BD Horizon; Cat# 555518, clone B159, 1:60 dilution
11. PE anti-human CD15 (SSEA-1); Biolegend; Cat# 301906, clone HI98, 1:20 dilution
12. BV650 Mouse Anti-Human CD11b; BD OptiBuild; Cat# 742640, clone ICRF44, 1:500 dilution
13. BV750 Mouse Anti-Human CD19; BD OptiBuild; Cat# 747161, clone SJ25CI, 1:500 dilution

Validation

1. anti-SRSF3 - validation statement on the manufacturer website: suitable for Flow Cytometry (Intra), ICC/IF; reacts with Human.
https://www.abcam.com/alexa-fluor-488-srsf3-antibody-epr16976-ab223455.html?productWallTab=ShowAll

2. anti-TRA2A - validation statement on the manufacturer website: suitable for IHC, IF; reacts with Human. This reagent is a Prestige Antibody, a group of antibodies developed and validated by the Human Protein Atlas (HPA) project (www.proteinatlas.org). As a result, they are supported by the most extensive characterization in the industry.
https://www.sigmaaldrich.com/BR/pt/product/sigma/hpa054018

*The antibodies described below are routinely used for the phenotypic characterization of human immune cells at the Flow Cytometry Facility in the A.C. Camargo Hospital. In addition to the appropriate validation provided by the manufacturer, the antibodies have been extensively tested at the Facility both alone and in combination to assure a suitable specificity and resolution when evaluated in blood samples.
3. DC-SIGN - validation statement on the manufacturer website: suitable for Flow Cytometry; reacts with Human.
https://www.biolegend.com/en-us/search-results/pe-cyanine7-anti-human-cd209-dc-sign-antibody-7059?Clone=9E9A8

4. anti-CD3 - validation statement on the manufacturer website: suitable for Flow Cytometry, ICC, IHC-F; reacts with Human
https://www.biolegend.com/en-us/products/brilliant-violet-421-anti-human-cd3-antibody-7153?Clone=UCHT1

5. anti-CD14 - validation statement on the manufacturer website: suitable for Flow Cytometry; reacts with Human.
https://www.biolegend.com/en-us/search-results/apc-cyanine7-anti-human-cd14-antibody-12788?Clone=63D3

6. anti-CD45 - validation statement on the manufacturer website: suitable for Flow Cytometry; reacts with Human.
https://www.bdbiosciences.com/en-br/products/reagents/flow-cytometry-reagents/research-reagents/single-color-antibodies-ruo/buv805-mouse-anti-human-cd45.612891

7. anti-CD4 - validation statement on the manufacturer website: suitable for Flow Cytometry; reacts with Human.
https://www.bdbiosciences.com/en-eu/products/reagents/flow-cytometry-reagents/research-reagents/single-color-antibodies-ruo/bv605-mouse-anti-human-cd4.565998

8. anti-CD8 - validation statement on the manufacturer website: suitable for Flow Cytometry; reacts with Human.
https://www.bdbiosciences.com/ko-kr/products/reagents/flow-cytometry-reagents/research-reagents/single-color-antibodies-ruo/bv650-mouse-anti-human-cd8.563821#

9. anti-CD25 - validation statement on the manufacturer website: suitable for Flow Cytometry; reacts with Human.
https://www.bdbiosciences.com/en-au/products/reagents/flow-cytometry-reagents/research-reagents/single-color-antibodies-ruo/buv563-mouse-anti-human-cd25.612918

10. anti-CD56 - validation statement on the manufacturer website: suitable for Flow Cytometry; reacts with Human.
https://www.bdbiosciences.com/en-br/products/reagents/flow-cytometry-reagents/research-reagents/single-color-antibodies-ruo/apc-mouse-anti-human-cd56-ncam-1.555518

11. anti-CD15 - validation statement on the manufacturer website: suitable for Flow Cytometry; reacts with Human.
https://www.biolegend.com/en-us/products/pe-anti-human-cd15-ssea-1-antibody-713?Clone=HI98

12. anti-CD11b - validation statement on the manufacturer website: suitable for Flow Cytometry; reacts with Human.
https://www.bdbiosciences.com/en-us/products/reagents/flow-cytometry-reagents/research-reagents/single-color-antibodies-ruo/bv650-mouse-anti-human-cd11b.742640

13. anti-CD19 - validation statement on the manufacturer website: suitable for Flow Cytometry; reacts with Human.
https://www.bdbiosciences.com/en-us/products/reagents/flow-cytometry-reagents/research-reagents/single-color-antibodies-ruo/bv750-mouse-anti-human-cd19.747161

Human research participants

Policy information about studies involving human research participants

Population characteristics

An 93-patient cohort with HNSCC obtained from the oral cavity (n = 71 patients), larynx (n = 11 patients), and oropharynx (n = 1 patient) from October 2009 to December 2021 was included in this study. The main clinical and pathological characteristics are summarized in Supplementary Table 1.

Recruitment

The HNSCC participants were included during the indicated period, and the criteria for patient selection was no treatment before samples collection. FFPE tissue, buffy coat and saliva samples were retrospectively obtained at the Valparaiso University archive and at the Biobanks of the Faculty of Medicine of Jundiai and ICESP. Additionally, a group of HNSCC buffy coat samples was prospectively collected at ICESP and A.C. Camargo Hospital according to the institutional availability. We are not aware of any biases that may impact the results. There was no participant compensation.

Ethics oversight

This study was approved by the Ethics Committees of Carlos Van Buren Hospital (Process 121), University of Valparaíso (Process CB051-14), University of São Paulo Academic Biobank of Research on Cancer, Centro de Investigação Translacional em Oncologia, Instituto do Câncer do Estado de São Paulo (ICESP) (Protocol CAEE 30658014.1.1001.0065), Faculty of Medicine of Jundiai (Protocol CAEE 45091715.1.0000.5412), and A.C. Camargo Hospital (Protocol 2532/18B).

Note that full information on the approval of the study protocol must also be provided in the manuscript.

Flow Cytometry

Plots

Confirm that:

- [x] The axis labels state the marker and fluorochrome used (e.g. CD4-FITC).
- [x] The axis scales are clearly visible. Include numbers along axes only for bottom left plot of group (a 'group' is an analysis of identical markers).
- [x] All plots are contour plots with outliers or pseudocolor plots.
- [x] A numerical value for number of cells or percentage (with statistics) is provided.

Methodology

Sample preparation

Cell suspensions were stained for surface markers using anti-CD45-BUV805 (BD Horizon, USA; clone HI30; 1:500 dilution), anti-CD3-BV421 (Biolegend, USA; clone UCHT1; 1:300 dilution), anti-CD4-BV605 (BD Horizon, USA; clone SK3; 1:300 dilution), anti-CD8-BV650 (BD Horizon, USA; clone RPA-T8; 1:500 dilution), anti-CD25-BUV563 (BD Horizon, USA; clone 2A3; 1:500 dilution), anti-CD56-APC (BD Horizon, USA; clone B159; 1:60 dilution), anti-CD19-BV750 (BD OptiBuild; clone SJ25CI; 1:500

dilution), anti-CD209-PE-Cy7 (Biolegend, USA; clone 9E9A8; 1:20 dilution), anti-CD14-APC-Cy7 (Biolegend, USA; clone 63D3; 1:200 dilution), anti-CD15-PE (Biolegend, USA; clone HI98; 1:20 dilution) and anti-CD11b-BV650 (BD OptiBuild; clone ICRF44; 1:500 dilution) monoclonal antibodies by incubation for 30 min with antibody solutions, followed by washes. TRA2A antibody (Sigma-Aldrich, USA; polyclonal; 1:100 dilution) was labeled by Zenon™ Alexa Fluor™ 750 Rabbit IgG Labeling Kit (Thermo Scientific, USA), as recommended by the manufacturer. The cells were permeabilized using the BD Pharmingen™ Transcription Factor Buffer Set (BD Biosciences, USA) for 40 min at 4°C and further stained with anti-SRSF3-FITC (Abcam; clone EPR16976; 1:20 dilution) and anti-TRA2A-Alexa Fluor 750 for 40 min at 4°C followed by washes. Two PBMC samples from HNSCC patients were included as controls for surface markers. Fc block was used to prevent non-specific antibody binding.

Instrument

FACSymphony equipment (BD Biosciences, USA)

Software

FlowJo v10.8 software (BD Biosciences, USA)

Cell population abundance

The buffy coat samples are primarily composed of leukocytes and granulocytes (Sutton, D. W., Chen, P. C. & Schmid-Schönbein, G. W. Cell separation in the buffy coat. Biorheology 25, 663-673, doi:10.3233/bir-1988-25406 (1988))

Gating strategy

Total leukocytes were first gated on a side scatter (SSC-A)/CD45 plot to define lymphocytes and myeloid cells. The lymphocytes were then gated on the CD19+ (B cells), CD56+CD3- (NK cells) and CD56-CD3+ (T lymphocytes) populations. These were further gated on the CD4+, CD8+ and CD4+CD25+ (activated T lymphocytes) subsets. The myeloid cells were separated by CD11b expression and further phenotyped according to CD209 (dendritic cells), CD15 (neutrophils) and CD14 (monocytes) expression. For more details, please see Supplementary figure 7.

☒ Tick this box to confirm that a figure exemplifying the gating strategy is provided in the Supplementary Information.
